# Laboratory-based 3D X-ray standing-wave analysis of nanometre-scale gratings

**DOI:** 10.1107/S1600576724007179

**Published:** 2024-08-19

**Authors:** Ksenia Matveevskii, Konstantin V. Nikolaev, Roberto Fallica, Detlef Beckers, Milen Gateshki, Alexander Kharchenko, Bart Spanjer, Alexander Rogachev, Sergey Yakunin, Marcelo Ackermann, Igor A. Makhotkin

**Affiliations:** a University of Twente Drienerlolaan 5 Enschde 7522 NB The Netherlands; b NRC Kurchatov Institute Moscow Russian Federation; c imec Leuven Belgium; d Malvern Panalytical BV, Almelo, The Netherlands; Argonne National Laboratory, USA

**Keywords:** X-ray standing waves, grazing-incidence X-ray fluorescence, laboratory metrology, many-beam dynamical diffraction theory

## Abstract

The increasing structural complexity and downscaling of modern nanodevices require continuous development of structural characterization techniques that support R&D and manufacturing processes. This work explores the capability of laboratory characterization of periodic planar nanostructures using 3D X-ray standing waves as a promising method for reconstructing atomic profiles of planar nanostructures. The non-destructive nature of this metrology technique makes it highly versatile and particularly suitable for studying various types of samples. Moreover, it eliminates the need for additional sample preparation before use and can achieve sub-nanometre reconstruction resolution using widely available laboratory setups, as demonstrated on a diffractometer equipped with a microfocus X-ray tube with a copper anode.

## Abstract 2

The 3D X-ray standing wave (XSW) technique is a new method for characterization of the 3D atomic profiles of planar periodic nanostructures, like nanometre-sized gratings or pillar arrays. The laboratory 3D XSW analysis of TiN gratings with nanometre-sized pitches is demonstrated here.

## 1. Introduction

The constant improvement in nanolithography and semiconductor technologies not only leads to a continuous decrease in the size of electronic devices but also allows an increase in the complexity of the structures (Mallik *et al.*, 2017 ▸). This progression can be observed in the evolution of transistors from planar configurations to fin field-effect transistors (Maurya & Bhowmick, 2022 ▸) and to even more complex structures like gate-all-around (Bhol *et al.*, 2022 ▸), as well as other structures such as black silicon (Fan *et al.*, 2021 ▸) and plasmonic arrays (Kasani *et al.*, 2019 ▸) and structures used for electrochemical energy conversion and storage (Zhao & Lei, 2020 ▸). Consequently, precise and accessible metrology methods for the geometrical characterization of such structures, *e.g.* critical dimension (CD) and morphology, are required at each stage of the manufacturing of such devices.

For characterizing planar nanostructures, numerous well established methods exist, such as electron- and atomic-probe microscopy (TEM, SEM, AFM) (Griffiths *et al.*, 2014 ▸; Belu *et al.*, 2016 ▸; Yan *et al.*, 2010 ▸; Hübschen *et al.*, 2016 ▸). However, these techniques have their limitations (Hässler-Grohne *et al.*, 2011 ▸). For example, TEM (transmission electron microscopy) requires extensive sample preparation (Griffiths *et al.*, 2014 ▸) that is costly and time consuming, while others like SEM (scanning electron microscopy) and AFM (atomic force microscopy) have physical limits of purely surface sensitivity. Furthermore, while microscopy techniques provide extensive information about small areas (about up to 100 µm^2^) (Allars *et al.*, 2021 ▸) of the sample, they lack the ability to efficiently provide ensemble information for tens of millimetres of the structures, which can be crucial for certain applications where averaged characteristics of the sample hold more significance than precise characterization of specific parts.

In this context, techniques based on photon scattering offer an interesting alternative. These techniques are non-destructive and can potentially be performed using regular in-laboratory X-ray sources, such as diffractometers, without requiring extensive sample preparation. They also provide averaged (over tens of mm^2^ large) area information about sample features. Photon scattering techniques, including X-ray diffraction, small-angle X-ray scattering, X-ray fluorescence (XRF) and optical-based techniques, are widely used in industry and laboratory settings for the characterization of nanoparticles and thin films (Hübschen *et al.*, 2016 ▸; Chu & Liu, 2000 ▸; Pauw, 2013 ▸).

The series of angle-resolved XRF techniques is specifically interesting for modern nanotechnology, including semiconductor engineering, since it can provide information about the atomic profile of periodic planar 3D nanostructure. Soltwisch *et al.* (2018 ▸) successfully demonstrated the application of this technique for reconstruction of an Si_3_N_4_ lamellar grating geometry using X-ray florescence measured at various grazing and azimuthal angles of incidence (referred to here as an XRF map) on a synchrotron source with the aid of the finite-element method (FEM). Their results showed the good agreement between the X-ray standing wave technique, SEM measurements and nominal parameters of the structure. The application of X-ray standing waves for thin-film analysis (‘1D structures’) for semiconductor devices has already been reported (Skytt *et al.*, 1994 ▸; Schmitt *et al.*, 1992 ▸).

The technique relies on creating a 3D X-ray standing wave (or XSW) inside the sample, which excites secondary processes such as XRF. The intensity of the XRF signal is enhanced by the antinodes of the XSW and suppressed by the nodes. By changing the angle of the incoming beam, the configuration of the XSW is altered, thereby modulating the resulting XRF signal (Zegenhagen & Kazimirov, 2013 ▸). In contrast to the traditional XSW technique in 1D samples, in the case of laterally periodic structures we use a combination of incident and azimuthal angle scanning to scan the nodes of the XSW in three dimensions. Therefore, we refer to the method reported here as the 3D XSW technique. The incidence rotation provides the characterization as a function of depth, similarly to the technique applied to thin films, but the azimuthal rotation of the sample with respect to the incident beam modulation of diffraction occurring in the complex structured layer also enables characterization of the in-plane geometry.

The advantages of this technique for studying nanoscale samples include the general benefits of X-ray techniques, such as non-destructiveness, potential for high speed and the possibility of reconstructing atomic profiles of 3D samples.

Apart from synchrotron installations, the feasibility of the 3D XSW method has been demonstrated on optimized laboratory setups based on a laser-produced plasma source (Baumann *et al.*, 2021 ▸). The study has shown the applicability of 3D XSWs in laboratory conditions, potentially enhancing the capabilities of in-laboratory metrology by offering non-destructive measurements suitable for atomic profile reconstruction of structured 3D samples without extensive sample preparations. To analyse these measurements, the technique often employs the FEM-based solvers of the Maxwell equations (Pomplun *et al.*, 2007 ▸) to compute the XSW and simulate the resulting intensity of characteristic X-ray fluorescent radiation.

The goal of this work is to investigate whether 3D XSW measurements can reasonably be performed on a laboratory diffractometer equipped with a microfocus X-ray tube with a copper anode. We also aim to estimate the feasibility of such measurements for arrays of periodic nanoscale structures used in semiconductor manufacturing and establish the requirements for the laboratory setup.

For this study, a set of nanogratings was used. The samples followed a typically industrially relevant design and manufacturing process and were not specially prepared with specific requirements from metrology. The samples were designed as periodic lines of TiN rectangular gratings.

Even though the samples were rather complex, with the right instruments we successfully characterized them with an estimated level of precision close to the industrial standard. 3D XSW measurements were performed at the Langmuir beamline of the Kurchatov synchrotron radiation source as a reference and with a Malvern Panalytical Microfocus copper-tube laboratory diffractometer.

## 2. Materials and methods

### 2.1. Nanograting sample fabrication

Samples of dense arrays of lines of pitch 32 nm were fabricated using a previously developed module for back-end-of-line metallization (De Simone *et al.*, 2021 ▸). Initially, a stack of SiO_2_/TiN/SiO_2_ (of thickness 20 nm/15 nm/15 nm, respectively) was deposited on top of a blank silicon wafer of 300 mm diameter. A sacrificial carbon hardmask of 35 nm was subsequently deposited on top of the stack. A 10 nm glassy underlayer was spin coated on top of the carbon layer. Finally, a negative tone metal–oxide resist, 22 nm thickness, was spin coated and patterned using extreme ultraviolet lithography (13.5 nm wavelength) in imec’s NXE3400B scanner using a pitch 32 nm vertical lines/spaces mask layout and a customized vertical dipole-like illumination. The dose of EUV light was varied in steps so as to obtain line/space arrays of varying critical dimensions, which explains how samples were extracted from die of different CD but equal pitch. After exposure and development, the photoresist pattern was transferred into the underlying layers using an integrated process in a TEL Tactras SCCM tool that etched through the titanium nitride layer, landing on the bottom silicon oxide layer. All other sacrificial layers were stripped in the process, except for the topmost silicon oxide layer which partially remained as a capping on top of the nitride lines. The wafer was subsequently diced for the purpose of X-ray characterization.

Each nanograting measured 15 nm in height, with a periodicity (pitch) of 32 nm, on the top of the SiO_2_ layer. The pattern covered the entire sample surface (∼13.2 × 8.6 mm^2^). All samples had a similar pitch of 32.1 ± 0.3 nm, although chips from different sets exhibited a different TiN line width (referred to as the CD) and line width roughness (LWR) of the grating. Two chips in total were analysed: one from each of two different triplet sets with line widths of 14.9 and 15.87 nm, analysed at the synchrotron and laboratory microfocus setups, respectively. These samples are denoted A and B, respectively.

### 2.2. Microscopy measurements of the sample

Top-down scanning electron microscopy inspection of the wafer was performed using a Hitachi critical-dimension scanning electron microscope (CD-SEM) using an acceleration voltage of 800 V, 0.8 nm per pixel resolution and a field of view of 819 nm^2^. Computational metrology to determine the average linewidth critical dimension was carried out using the tool’s inline image processing algorithms. Afterwards, a cutout ‘lamella’ of the line/space array was prepared using a Helios dual focused ion and electron beam tool and transferred to a Tecnai transmission electron microscope where an electron beam (300 kV acceleration) was used to inspect the sample in both bright-field and dark-field mode.

To gain initial insights into the grating profile, a cross-sectional transmission electron microscopy measurement was performed. It was discovered that, due to residuals from the etching process, the samples had a more complex geometry than initially designed. Some SiO_2_ was left unetched, creating a bulb-like structure at the top and an overetched valley at the bottom. Additionally, the grating exhibited a visible wall incline and a thin layer of residual SiO_2_ on the walls.

### 2.3. Measurement setup

When considering the requirements for the experimental setup suitable for 3D XSW measurements, firstly, the sample holder or the beam source should be adjustable in both azimuthal and incident angles so that modulation of the XSW in both in-plane and depth directions is possible. Another important requirement is that the optics should condition the beam to a pencil shape to be ‘parallel’ in both directions and have the lowest possible beam divergence. The source should also be intense enough to perform the measurements in a reasonable time. The beam width should be less than the width of the sample where the pattern is homogeneous.

#### 2.3.1. Synchrotron setup

The Langmuir synchrotron beamline has a monochromatic beam with 13 keV photon energy, with energy resolution ∼5 eV and with a direct beam integrated flux of 2 × 10^8^ c.p.s. The sample holder allows adjustment in six axes. The energy-dispersive SSD detector VORTEX X90 was used. The optical system consists of a double-crystal monochromator, collimator slits, a grazing-incidence X-ray mirror for high harmonic filtration and another set of collimator slits, allowing for an estimated beam divergence of less than 4′′ vertically and 12′′ horizontally. The measurements were performed with the sample exposed to the air.

#### 2.3.2. Microfocus setup

An Empyrean diffractometer from Malvern Panalytical was equipped with a microfocus X-ray tube with a copper anode. A graded multilayer mirror was used to collimate the incident beam in both directions. The beam size in the sample position was around 100 µm and the divergence was estimated to be 72′′. For the XRF measurements, an energy-dispersive SDD detector (KETEK VITUS H150) with 150 mm^2^ chip area and a Be window was used.

### 2.4. XRF acquisition

In both experiments, XRF spectra were measured for all elements excited by the respective source at different points. Then the integrated signal from the Ti *K* line was deconvoluted using the open-source *PyMCA* package (Solé *et al.*, 2007 ▸).

### 2.5. Modelling using MBDDT

For sample characterization, the many-beam dynamical diffraction theory (MBDDT) formalism has been used. This method enables us to simulate XRF using the Sherman equation with a semi-analytical calculation of the field components provided by diffraction theory (Nikolaev *et al.*, 2020 ▸), which allows for a great reduction of computation time compared with precise methods like the FEM-based solvers of the Maxwell equations.

The model was fitted iteratively with an initial model based on the TEM data and fine fitting of the parameters being performed by minimization of the χ^2^ function.

## 3. Physics of 3D XSW signal formation

The 3D XSW method is based on the measurement of fluorescent intensity modulation, caused by an XSW in the sample which excites the fluorescent atoms in the XSW’s antinode position.

The intensity of XRF radiation excited by an XSW for a given element *e* at a given pair of angles depends on the likelihood of the modulated electromagnetic field to excite fluorescent atoms and can be described by the equation (Jiang *et al.*, 2020 ▸; Nikolaev *et al.*, 2023 ▸)
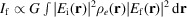
where *G* is a factor that depends on the geometrical configuration of the instrument and element-dependent parameters (such as the ionization cross section of the atoms), ρ_*e*_ is the fluorescent-atom concentration of element *e*, and 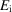 and 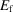 are the near field (NF) induced by the incident and fluorescent radiation, respectively (Hönicke *et al.*, 2010 ▸). When the scattering geometry is far from that required for dynamic effects, the near field is well approximated by a decaying exponential. This is consistent with the Beer–Lambert law. When the conditions for dynamic scattering such as diffraction or total external reflection are fulfilled, the XSW is formed and the near fields take a complex spatial configuration that gives rise to the modulation of the fluorescence intensity. For *E*_i_ this is due to the interference of the incident radiation, and for *E*_f_ this is due to the interference of the fluorescence photons – the Kossel effect. Thus the XSW method can be implemented in three different ways. The first is to set the scattering geometry to grazing incidence (GI) and normal detection, such that the incident radiation forms an XSW. The second is, *vice versa*, the grazing emission technique. The third is an exotic approach where both the incident radiation and the fluorescence photon induce XSWs (Tsuji *et al.*, 2000 ▸).

For the use of the 3D XSW technique two conditions should be fulfilled – the sample should be periodic and smooth enough that it would be able to form a stable out-going diffracted beam for the XSW, and the incoming beam should be parallel in all directions to provide sufficient resolution for the technique.

In the case of GI geometry, the XSW is formed by interference between the incident and diffracted beam appearing at low incident angles and exists in the presence of the combination of two strong effects – strong total external reflectance and diffraction. Varying the angle of the incident beam modulates the XSW configuration inside the sample and leads to changes in the measured fluorescent radiation intensity. While the change of incident angle 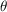 modulates the XSW in the depth direction due to the scattering of evanescent waves, the change of the azimuthal angle 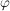 modulates the XSW in-plane due to diffraction. So, a combination of the measurements in both directions should be used to reconstruct the atomic profile of the 2D sample. Such measurements can take the form of a set of isolated scans done around different angles or full XRF maps. The field in the sample arises when the beam is in the diffraction condition and the diffracted beams interfere with reflected beams. The field is especially strong where the surface diffraction condition is strongest, producing the features described as the resonant lines (Nikolaev *et al.*, 2020 ▸). These lines arise when the nodes of the reciprocal lattice cross Ewald’s sphere and are similar to Kikuchi lines in single crystals (Baba-Kishi, 1998 ▸), although in our case their positions will not match the predicted positions for Kikuchi lines. A similar effect has been observed in reflection high-energy electron diffraction (RHEED) where observed intensity enhancements at certain incident angles were explained by certain resonant effects (Maksym & Beeby, 1981 ▸, Marten & Meyer-Ehmsen, 1985 ▸). As the mathematics behind the observed RHEED effect looks similar to that describing resonant effects in the case of 3D XSWs in GI geometry, a similar pattern is expected to be presented on measured XRF maps.

**Figure 1:**
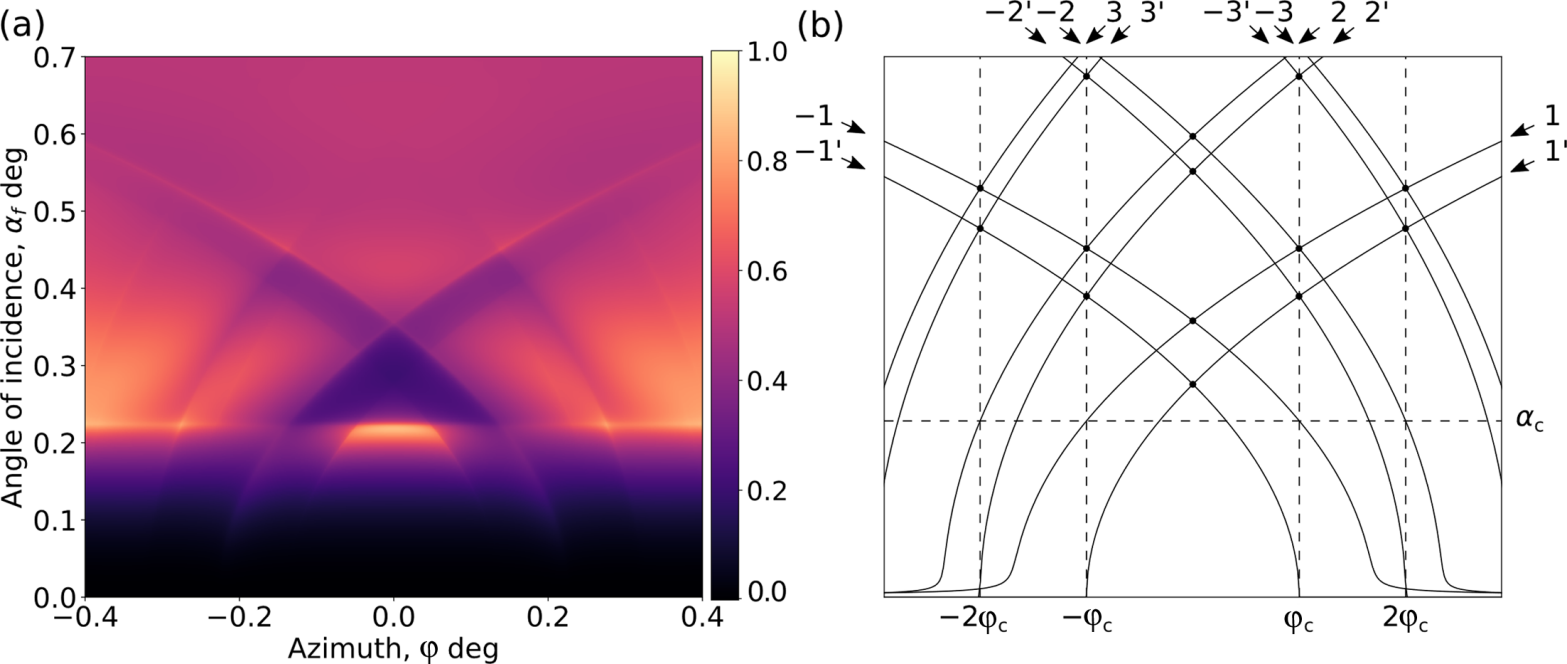
(*a*) 3D XSW map simulated using MBDDT on the basis of the simple grating model. (*b*) Several distinct features are present. α_c_ and φ_c_ indicate the critical incident and azimuthal angles; numbered lines indicates diffraction lines of *n*th diffraction order in a structured layer (…, −2, −1, 1, 2,…) and in vacuum (…, −2′, −1′, 1′, 2′,…).

To demonstrate this we simulated a 3D XRF map for an idealized square TiN grating profile with period 32 nm, width 16 nm and height 15 nm on top of an Si substrate (Fig. 1 ▸). The excitation radiation is consider to be of the Cu *K*α line and the resulting fluorescent emission was calculated for Ti. The fluorescence intensity within the map exhibits a significant dependence on both the incident angle and the azimuthal angle of the incoming beam. Notably, the map highlights several distinct features which are expected from our understanding of the theory:

Resonant lines calculated for the waves propagating inside the structured layer are marked with integers (*e.g.* −2, −1, 1, 2) and the lines that are above the sample are marked with primed integers (*e.g.* −2′, −1′, 1′, 2′). A straight horizontal line at an angle equal to α_c_ is clearly visible, resembling the angle of total external reflection. We can define the critical azimuthal angle 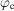 as the angle where the first-order diffraction line crosses the azimuthal angle line.

In the geometry when the incoming beam is far from parallel to the grating lines, the XSW diffraction effect becomes small, so the modulated near field in the sample is based only on the interference of incoming and reflected beams, and loses the sensitivity to the lateral structure. The sample thus can be treated as a thin film with averaging of the layer structures. In this approximation (named the effective medium approximation), the structured layer of the sample is treated as a layer of one diluted material with the density and dielectric permittivity averaged across the structure and ambient. To explain these features we consider the field modulated at different angles.

From the thin-film study, the critical incident angle is the angle at which total external reflection at the film is achieved and evanescent waves and thus XSWs can be formed. Analogously, for the grating, the critical incident angle 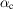 is defined as the angle of total external reflection in a geometry far from parallel and equals
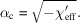
Here 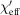 is the real component of the average dielectric susceptibility for the structured layer, which in the case of X-ray wavelengths has a negative sign.

The resonance lines can be described by the spherical dispersion (Mikulík & Baumbach, 1999 ▸) for the *m*th order of diffraction (assuming elastic scattering and invariance of the wavevector as a function of depth):
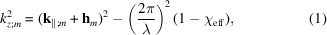
where 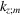 and 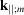 are components of the wavevector of the diffracted beam of *m*th order, 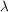 is the wavelength of the incoming beam, and 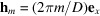 is the reciprocal lattice vector of the grating, with *D* the period of the grating.
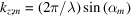
and
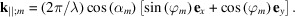


Then equation (1) can be multiplied by 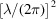 and written in the form
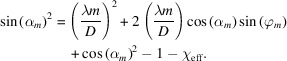


As in GI geometry the incident angle is small and 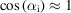, the angle coordinates of points on the diffraction line of *m*th order follow the equation
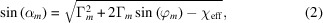
where 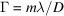.

As the pattern of the diffraction lines depends only on the wavelength and average dielectric susceptibility for the structured layer, it can be used for the angle alignment in the case of a known structure or provide information on the dielectric permittivity in the case of a well aligned setup.

Putting the value of the critical incident angle 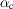 into equation (2), we can now define the characteristic value for the critical azimuthal angle 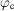, which characterizes the angle at which we can see changes in diffraction effects:
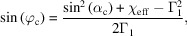
or as 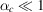,
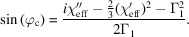


In the case of X-rays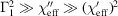. As a result, the critical azimuthal angle takes the form of
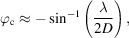
which shows that the real part of the critical azimuthal angle aligns with the first diffraction order.

Also from here we can see that the position at which the *m*th-order diffraction line crosses the critical incident angle follows the equation
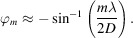


**Figure 2:**
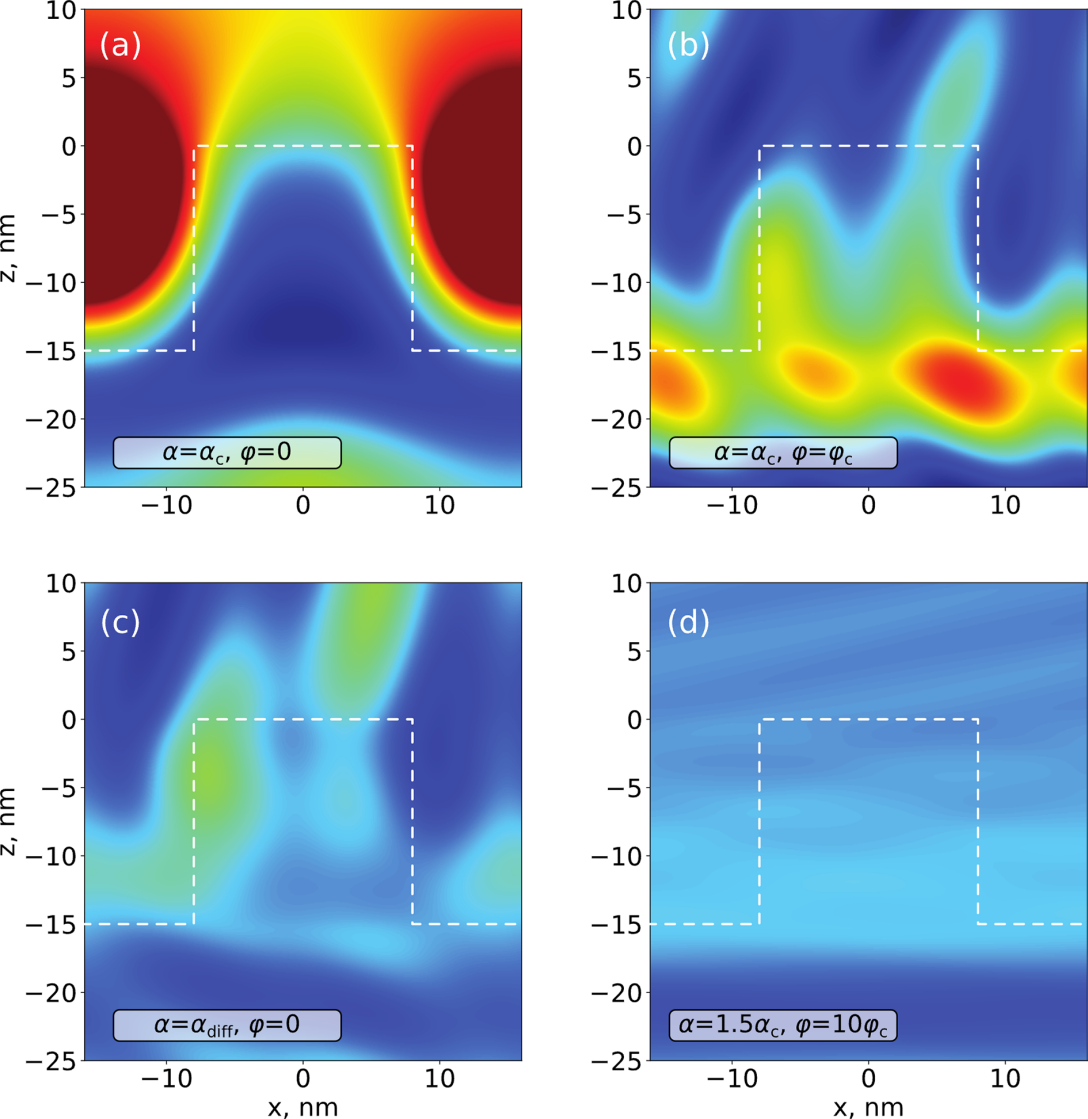
Near-field energy density penetration inside the model nanograting (as shown in Fig. 1 ▸) calculated at different configurations of the incident beam. (*a*) Incidence angle is critical and azimuthal angle is less than critical – the field does not penetrate deep inside the sample structure. (*b*) Incidence and azimuthal angles are critical – the field penetrates the sample even if the incident angle is relatively small. (*c*) Incident angle is higher than critical (at the position of crossing of −1st- and 1st-order diffraction lines) and beam is parallel to the grating lines – the field penetrates the sample. (*d*) Incident beam angle is higher than critical and beam is far from parallel to the grating – the field looks uniform in the in-plane direction, so the structured layer can be treated as a thin-film layer of average density.

Thus we can qualitatively explain features on the GI XRF map and attribute them to different angular orientations of the grating. The NF simulations (Fig. 2 ▸) at the different angle points of the XRF map for the simulated structure (Fig. 1 ▸) show that at an incidence angle where the is beam parallel to the lines of the grating [Fig. 2 ▸(*a*)] the NF does not yet penetrate deep inside the grating structure. After rotation of the beam in the azimuthal direction [Fig. 2 ▸(*b*)] the NF starts to penetrate inside the sample and have an asymmetrical structure. A similar situation occurs at incidence angles higher than critical [Fig. 2 ▸(*c*)] when the NF penetrates inside the sample. And if we go too far from parallel to the lines of the grating with the critical azimuthal angle 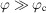 [Fig. 2 ▸(*d*)] the NF will become unified along the lateral direction, losing all sensitivity to the characteristic features, and the structured layer can be treated as a thin-film layer of average density (this approximation is usually named the averaged medium approximation).

All these features attributed to different angular orientations can be used as the starting point for fitting or for fast parameter characterization. In the centre, diffraction effects are the most prominent. As diffraction is modulated by the geometry of the grating, measurements done in this area have the highest sensitivity to the geometry and in-plane atomic profile of the grating. The geometry of diffraction lines modulated by the interface is only dependent on the pitch and the energy of the beam, and, as the beam energy and pitch are usually well defined parameters in the experiments and sample production, the fitting of the position of the these lines allows for refining the geometrical parameters of the experimental scheme. For the azimuthal incidence angle far from the first-order diffraction, the effective medium approximation works, making any measurements here only sensitive to the averaged structure.

While the physics of XSWs has several rather complex effects, we demontrate that performing the analysis using these principles on a set of asymptotes allows us to characterize the profile of the nanostructure.

## 4. Results

**Figure 3:**
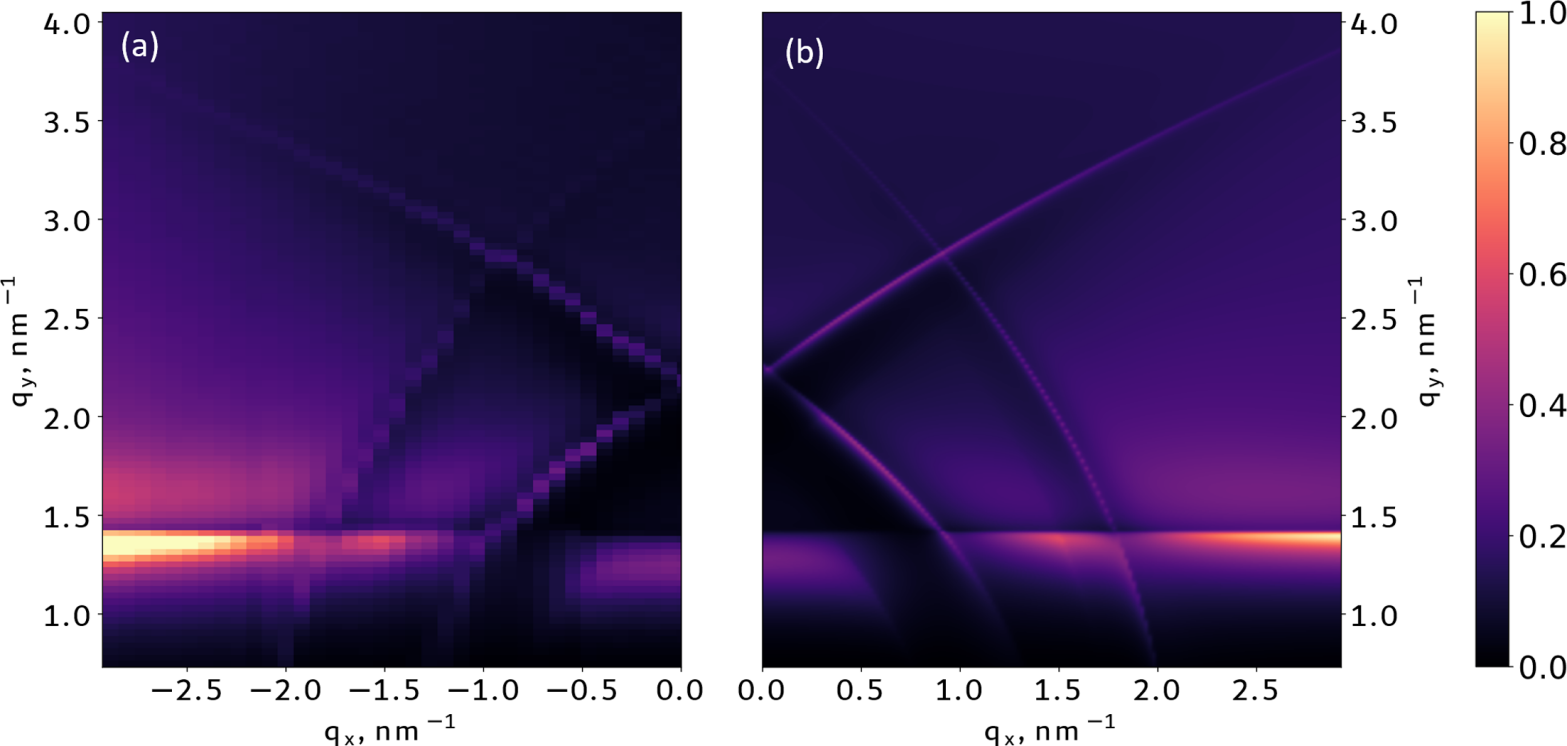
(*a*) The XRF map measured at the synchrotron facility. Features such as critical angle and diffraction lines forming the cross-shaped pattern in the centre as shown earlier are distinctly visible. (*b*) The simulated AR XRF map based on the parameters reconstructed using MBDDT.

XRF maps for samples measured at the synchrotron and cross sections measured using laboratory sources are shown in Figs. 3 ▸ and 4 ▸, respectively.

**Figure 5:**
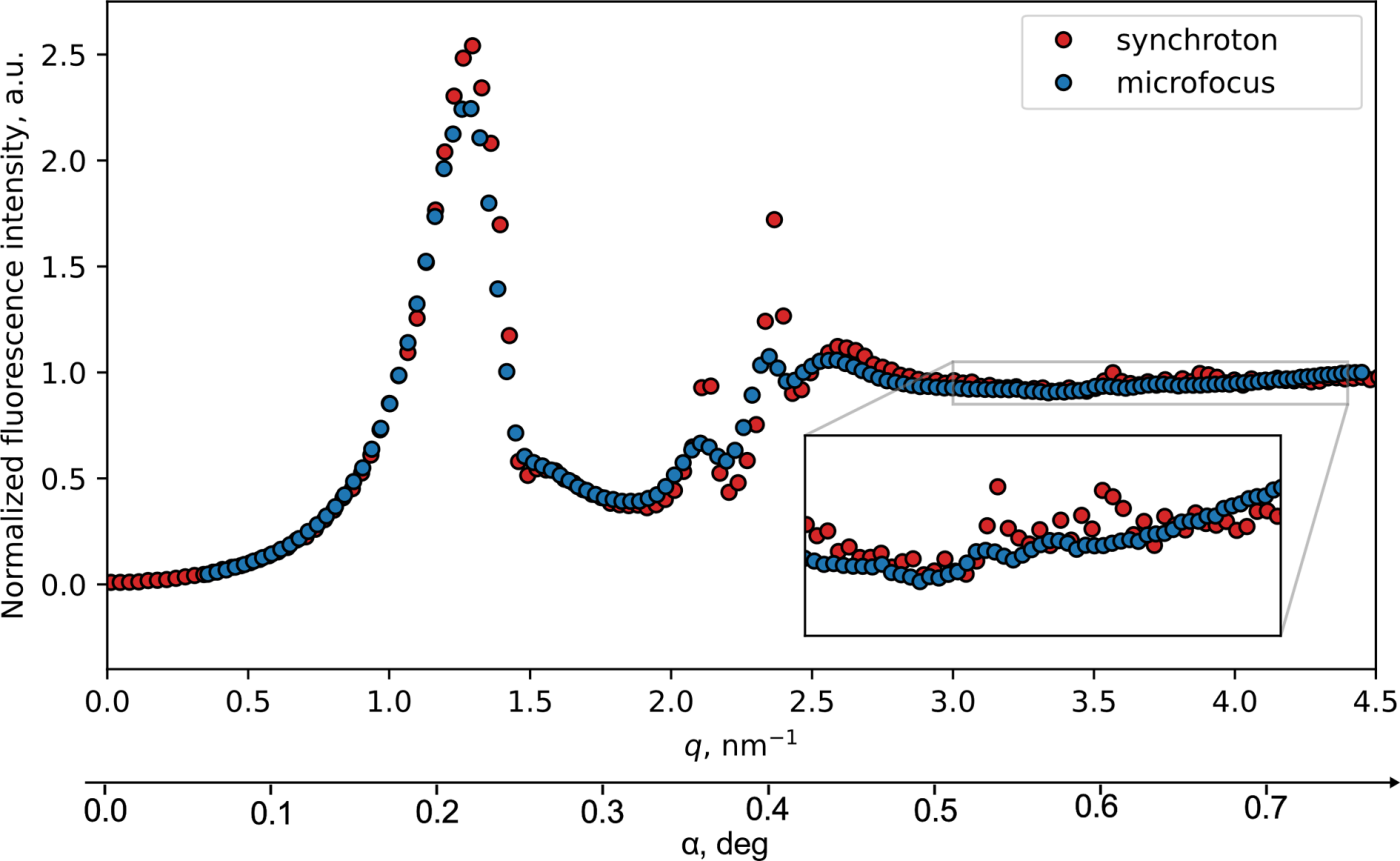
Cross sections measured on the synchrotron (red dots) and on the microfocus setup (blue dots). Axis values provided both in transfer vector magnitude *q* and incident angle 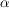 from microfocus measurements.

Upon comparing the cross sections obtained from the two setups (Fig. 5 ▸), it becomes evident that, following corrections for angle and beam divergence, the features are remarkably similar. Comparisons were done using the magnitude of the lateral momentum transfer vector 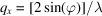 as coordinate, where 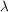 is the beam wavelength and φ the azimuthal angle, as data from different sources were measured at different angles. Minor disparities in peak intensities and positions are observed.

**Figure 6:**
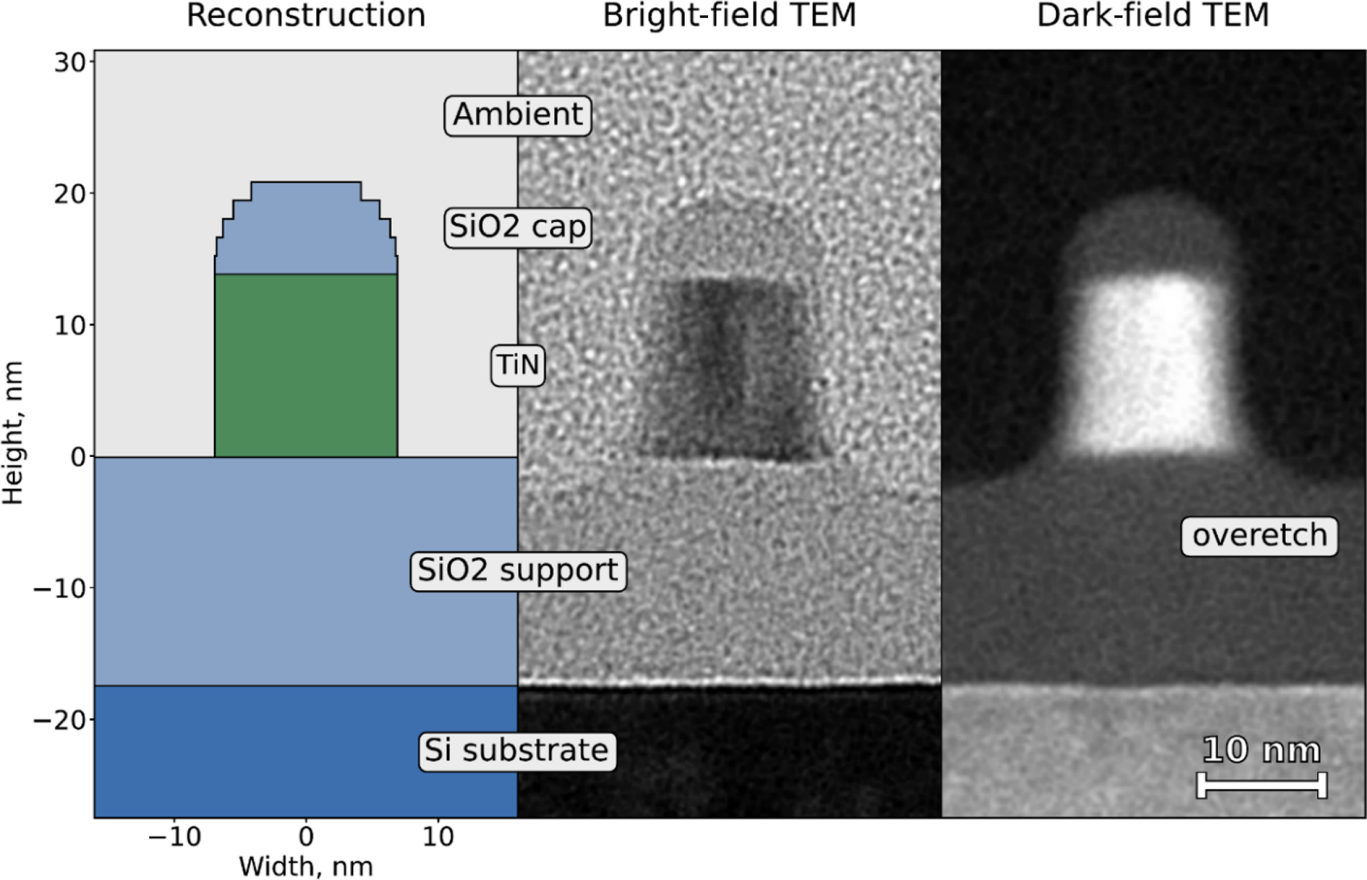
Final reconstructed profile of the nanograting based on the best fit of X-ray data in comparison with TEM images. Most of the features besides overetch are matched.

The initial-guess grating model was established on the basis of TEM scans. The model shown in Fig. 6 ▸ accounts for the dimensions of the TiN grating, including its height and width, the incline of its walls, the unetched SiO_2_ on the top and sidewalls, the width of the SiO_2_ support layer, and the presence of grooves. Numerical parameters such as the number of rectangular blocks used to define the curved geometry and the number of diffraction modes used for calculations were selected as the minimal amount at which a further increase will not significantly change the simulation.

**Figure 7:**
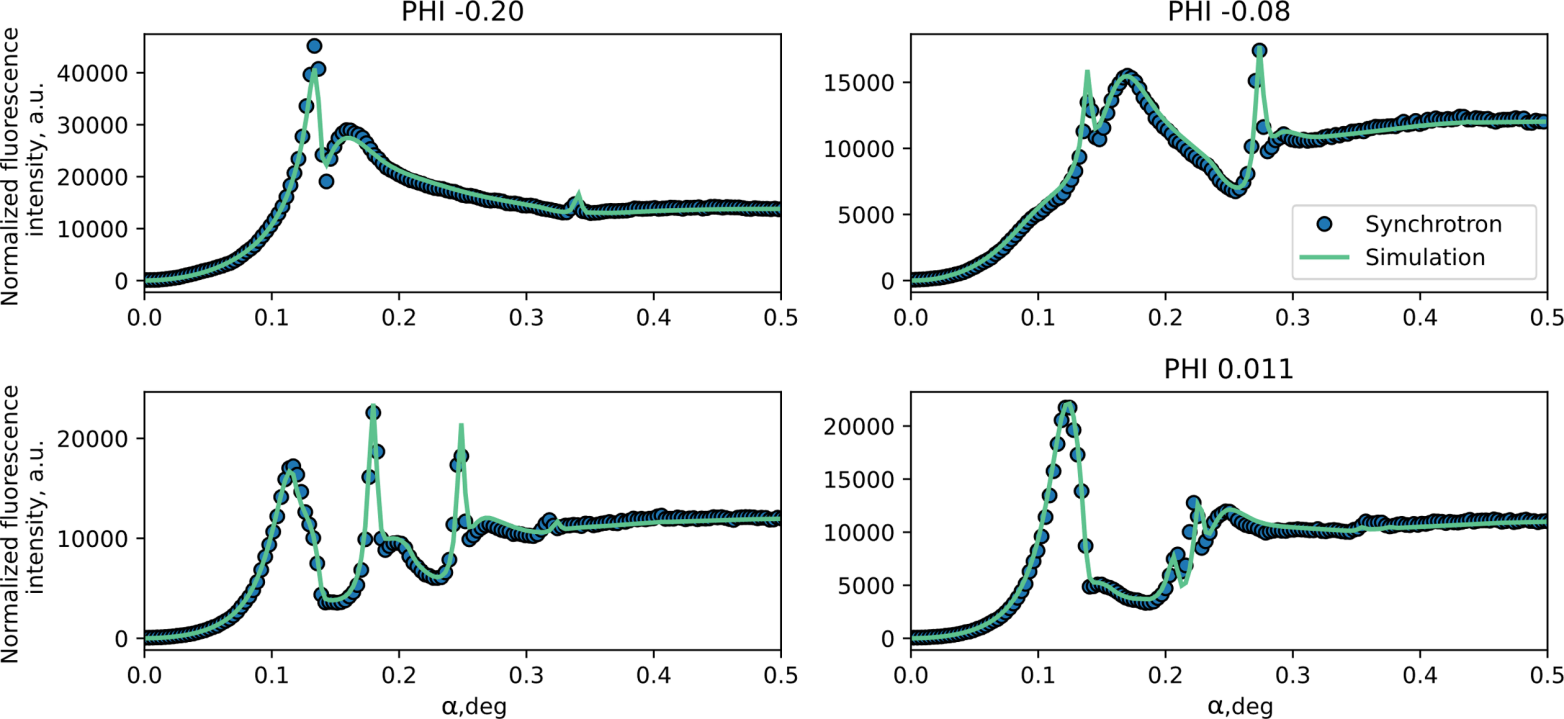
The separate cross sections for various fixed azimuthal angles measured on the synchrotron (blue dots) with applied corrections for beam geometry and signal simulated according to the fitted model parameters (green line).

**Figure 8:**
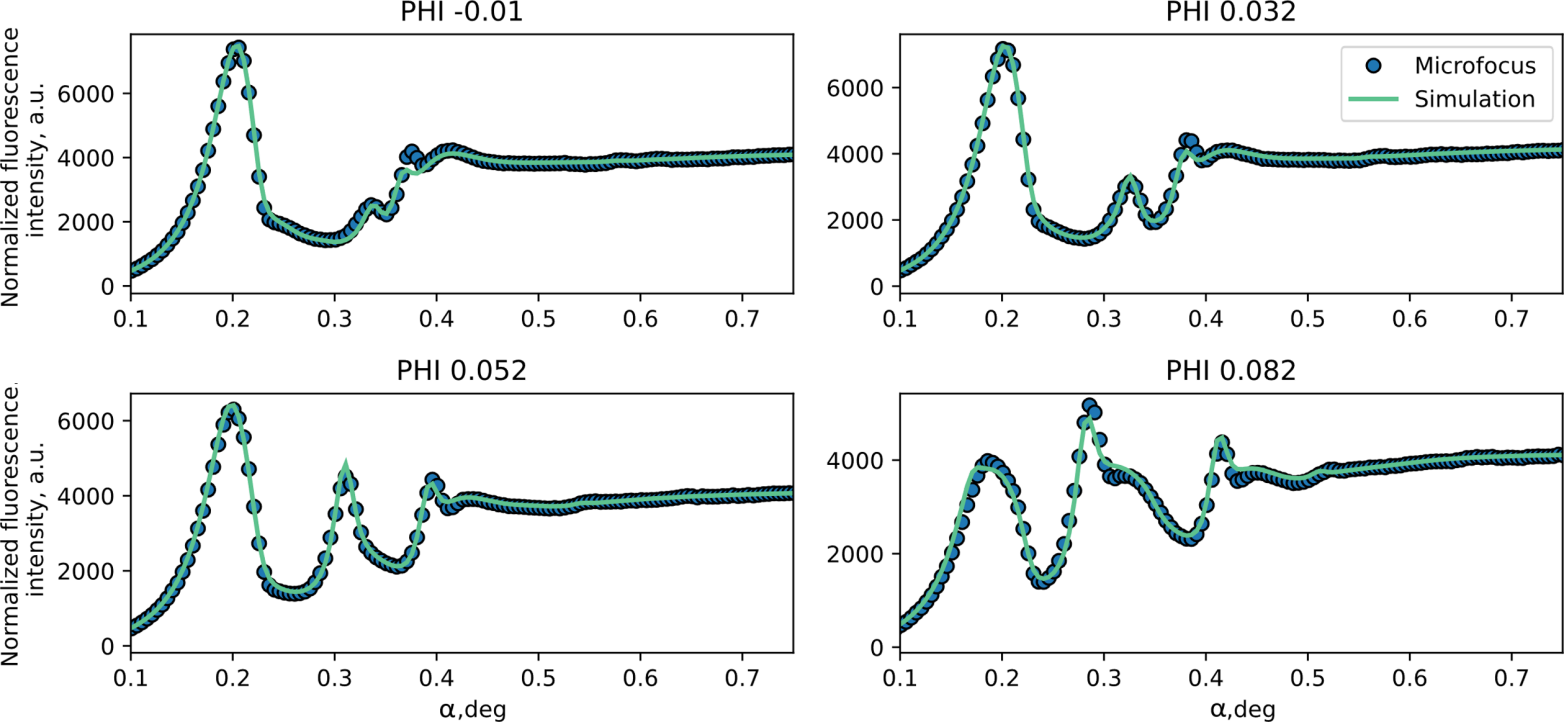
The separate cross sections for various fixed azimuthal angles measured on the microfocus setup (blue dots) with applied corrections for beam geometry and signal simulated according to the fitted model parameters (green line).

Reconstruction of the profiles was performed by fitting cross sections (Fig. 7 ▸) chosen from the measured XRF map on the synchrotron and measured cross sections for the microfocus source (Fig. 8 ▸). These cross sections were mostly selected in the central area where sensitivity to the grating geometry is the highest. The fitting was performed by means of minimization of the χ^2^ function of the cross section. The trust interval was estimated as the parameter values at which the χ^2^ function is two times larger than the χ^2^ function calculated for the best-fit model.

Upon fitting the data from Samples A and B, it was determined that the results are less sensitive to the presence of certain features, such as a slight wall incline, SiO_2_ on the sides and overetching, as fitting of selected cross sections allows these values to be equal to zero in the trust interval for the respective parameter. Reconstructed parameters for different samples display strong agreement in terms of geometry, while the TiN width increases from the sample measured on the synchrotron to the sample measured on the microfocus setup, in line with CD-SEM measurements. Note that the reconstructed values in the models, as well as the TEM measurements, are slightly smaller than the values obtained through CD-SEM. Furthermore, the TEM results for the sample measured on the synchrotron align well with the 3D XSW reconstructed model in values of the presented parameters (Tables 1 ▸ and 2 ▸).

Additionally, angular divergence of the beam has been taken into account during the calculation. It has been simulated as the convolution of the signal with a Gaussian function. From the fit, the divergence of the synchrotron beam is close to zero and was not possible to estimate, meaning that the beam is very close to parallel. The divergence of the microfocus setup is estimated to be around 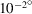. In previous measurements it was noted that with an angular divergence of ∼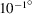 on a microfocus setup the XSW signal was too smoothed for reconstruction.

## 5. Discussion

By comparing the outcome achieved through various experimental configurations, it is evident that all reconstructed values, except for the grating width parameter, are similar with similar trust intervals. This consistency aligns with our expectations based on the deposition process. However, the trust intervals calculated for reconstructed parameters are only a rough estimation of the sensitivity of the parameter to change within the fitted model. The smaller trust intervals for some parameters of the microfocus setup compared with the fit of the synchrotron data can be attributed to the fact that the fit of the microfocus data is closer to the best possible fit.

The 3D XSW method provides ensemble information about the atomic profile of the planar nanostructures. The model of the atomic profile then holds essential information about the geometric parameters of the grating, such as width, CD and groove height. Comparing the 3D XSW with other measurement techniques, we observe that TEM and 3D XSW results tend to remain within their respective trust intervals. Notably, TEM imaging offers insights into the local sample geometry that the 3D XSW technique cannot discern with the selected set of cross sections, such as subtle wall inclinations or the presence of unetched SiO_2_ on walls or pedestals. Sensitivity to these parameters can be lower due to XRF being insensitive to Si in the used energy range and under ambient conditions. Conversely, TEM provides localized information, whereas the 3D XSW method averages signals across multiple periods. This discrepancy becomes evident by the fact that the TEM results for the grating period differ from both the CD-SEM and the 3D XSW imaging outcome.

When measurement of the intensity of the direct beam for the microfocus setup is not possible, we can estimate how fast the microfocus performs relative to the synchrotron setup. The time spent on measurement of one data point was 30 s for the microfocus and 100 s for the synchrotron setup. The maximum intensity of the Si peak is ∼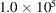 for the microfocus setup and ∼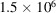 for the synchrotron. From this we can estimate that measurements performed on the synchrotron should be ∼5 times faster than one done on the microfocus setup for the same number of data points measured.

Grating width values derived from the TEM and 3D XSW methods generally appear smaller than those obtained through CD-SEM. This discrepancy can be attributed to several factors. Firstly, CD-SEM provides top-down imaging, resulting in width measurements primarily from the lower portion of the grating, where values are slightly elevated due to the wall slope of the structures. Additionally, neither TEM nor 3D XSW take into account the LWR. Moreover, the presence of unaccounted for SiO_2_ on the walls may potentially influence the reconstructed values when utilizing 3D XSWs.

Additionally, the technique can be performed in two different geometries: grazing incidence, where the beam is nearly parallel to the sample’s surface and the resulting XRF radiation is emitted perpendicular to the surface (the geometry that was used in this research), and grazing exit (GE), where the beam is perpendicular to the surface and the emitted XRF is parallel to the beam. Both geometries are described by the same mathematics and offer specific advantages and applications (Hönicke *et al.*, 2022 ▸). In the case of GI geometry, the beam spot is large, providing a stronger signal from a larger area of the sample, whereas in GE, the beam spot is relatively small, allowing scanning through the sample without mechanical movement, but with lower intensity. So a similar study performed in GE geometry can hold potential interest for the development of scanning metrology techniques in the laboratory or industrial environments.

## 6. Conclusion

Here we have demonstrated the laboratory implementation of the 3D XSW technique for the analysis of nanostructured gratings. We have derived a set of analytical formulas for describing the strong enhancement of the XRF signal that can be used for initial qualitative analysis of the 3D XSW data and building the initial-guess model. We have also demonstrated the results of the reconstruction of 3D XSW data measured using X-rays with 0.154 nm wavelength using the many-beam dynamical diffraction theory. The unique sensitivity of the 3D XSW technique to the ensemble information about the atomic profiles of periodic planar nanostructures provides ample possibility for the analysis of these structures to be fully revealed in follow-up research.

## Figures and Tables

**Figure 4:**
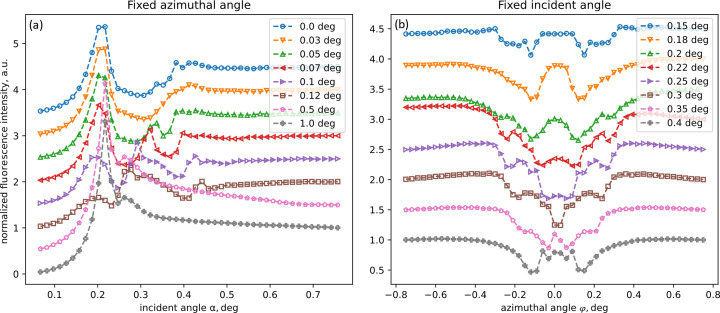
XRF cross sections measured on the microfocus in-laboratory source. The cross sections were measured at fixed azimuthal (*a*) and incident (*b*) angles.

**Table 1:** Measured and reconstructed parameters for the sample measured on the synchrotron obtained by different techniques

	CD-SEM	TEM	3D XSW
Period, nm	32.1 ± 0.3	33.1 ± 0.6	32.1 ± 0.3
Grating width, nm	14.9	12.1 ± 0.4	12.8 ± 0.9
Height of the grating, nm	–/15 by design	13.3 ± 0.2	13 ± 2
Support thickness, nm	–/20 by design	14.1 ± 0.4	18 ± 6
Cap thickness, nm	–	5.6 ± 0.2	8 ± 3
Pedestal height, nm	–	2.4 ± 0.5	0.0 ± 1.2

**Table 2:** Measured and reconstructed parameters for the sample measured on the microfocus setup obtained by different techniques

	CD-SEM	3D XSW
Period, nm	32.1 ± 0.3	32.1 ± 0.4
Grating width, nm	15.87	14.0 ± 0.6
Height of the grating, nm	–/15 by design	13.9 ± 1.1
Support thickness, nm	–/20 by design	18 ± 10
Cap thickness, nm	–	7.0 ± 1.5
Pedestal height, nm	–	0.0 ± 0.9
